# Supplementary material for: Peptidomics Analysis Reveals the Buccal Gland of Jawless Vertebrate Lamprey as a Source of Multiple Bioactive Peptides
Source: Mar Drugs. 2023 Jun 29;21(7):389. doi: 10.3390/md21070389 (PMC10381800; doi:10.3390/md21070389)
Supplement: Supplementary file 1 [file marinedrugs-21-00389-s001.zip › marinedrugs-2454142-supplementary/Supplementary Figures.pptx]

## Slide 1
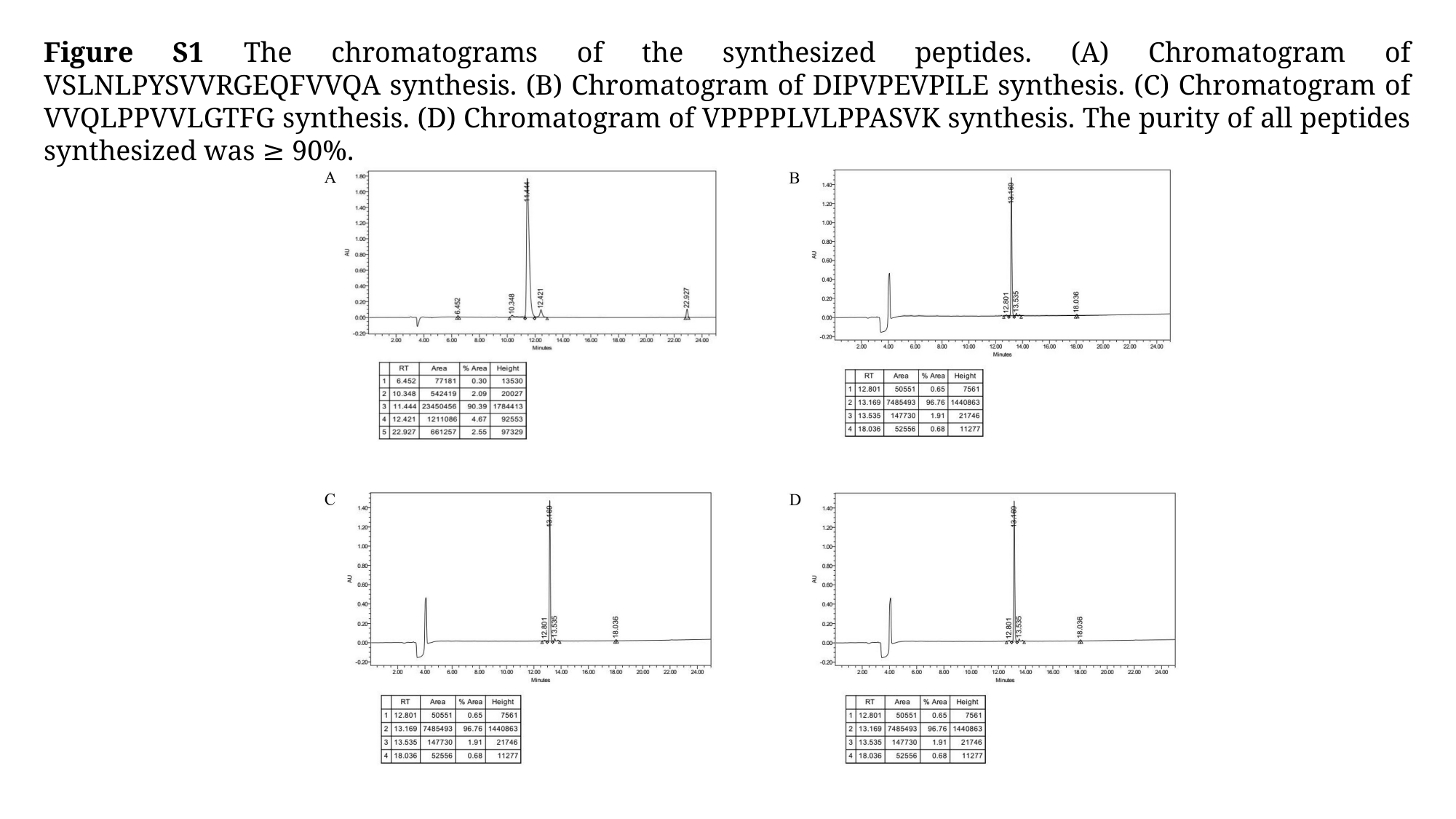

Figure S1 The chromatograms of the synthesized peptides. (A) Chromatogram of VSLNLPYSVVRGEQFVVQA synthesis. (B) Chromatogram of DIPVPEVPILE synthesis. (C) Chromatogram of VVQLPPVVLGTFG synthesis. (D) Chromatogram of VPPPPLVLPPASVK synthesis. The purity of all peptides synthesized was ≥ 90%.

## Slide 2
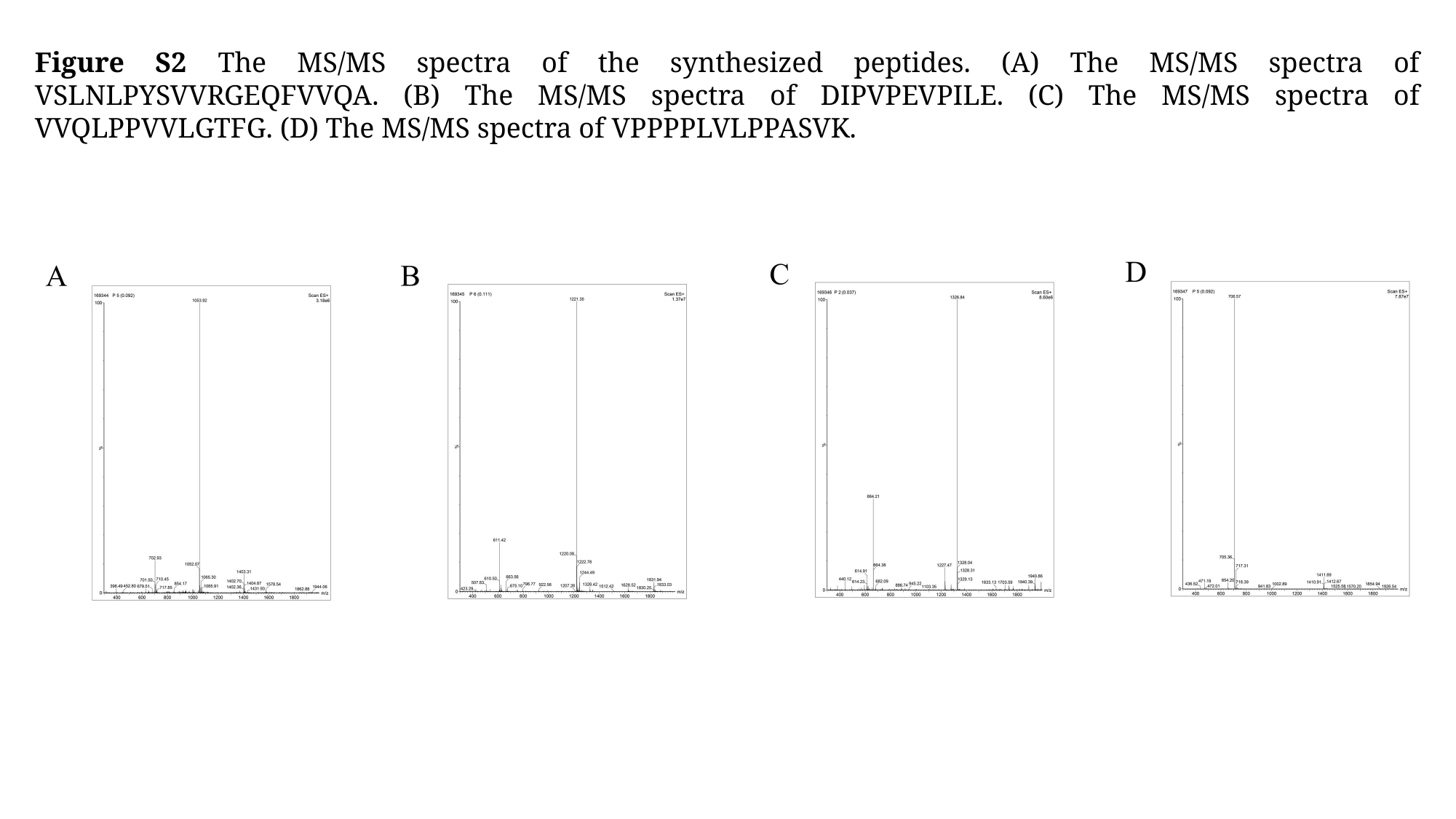

Figure S2 The MS/MS spectra of the synthesized peptides. (A) The MS/MS spectra of VSLNLPYSVVRGEQFVVQA. (B) The MS/MS spectra of DIPVPEVPILE. (C) The MS/MS spectra of VVQLPPVVLGTFG. (D) The MS/MS spectra of VPPPPLVLPPASVK.

## Slide 3
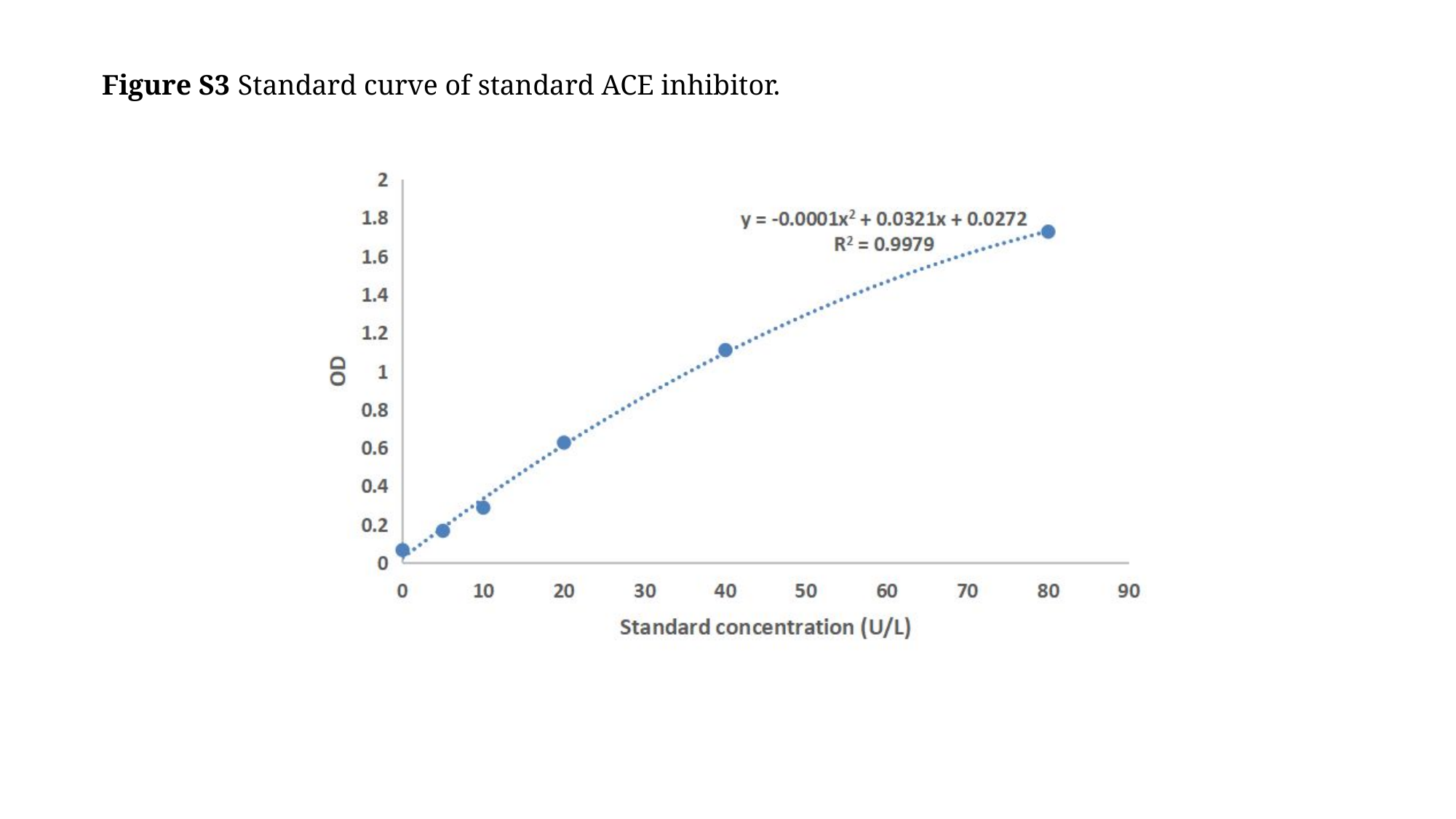

Figure S3 Standard curve of standard ACE inhibitor.
