# Supplementary figures and images for: Peptidomics Analysis Reveals the Buccal Gland of Jawless Vertebrate Lamprey as a Source of Multiple Bioactive Peptides
Source: Mar Drugs. 2023 Jun 29;21(7):389. doi: 10.3390/md21070389 (PMC10381800; doi:10.3390/md21070389)

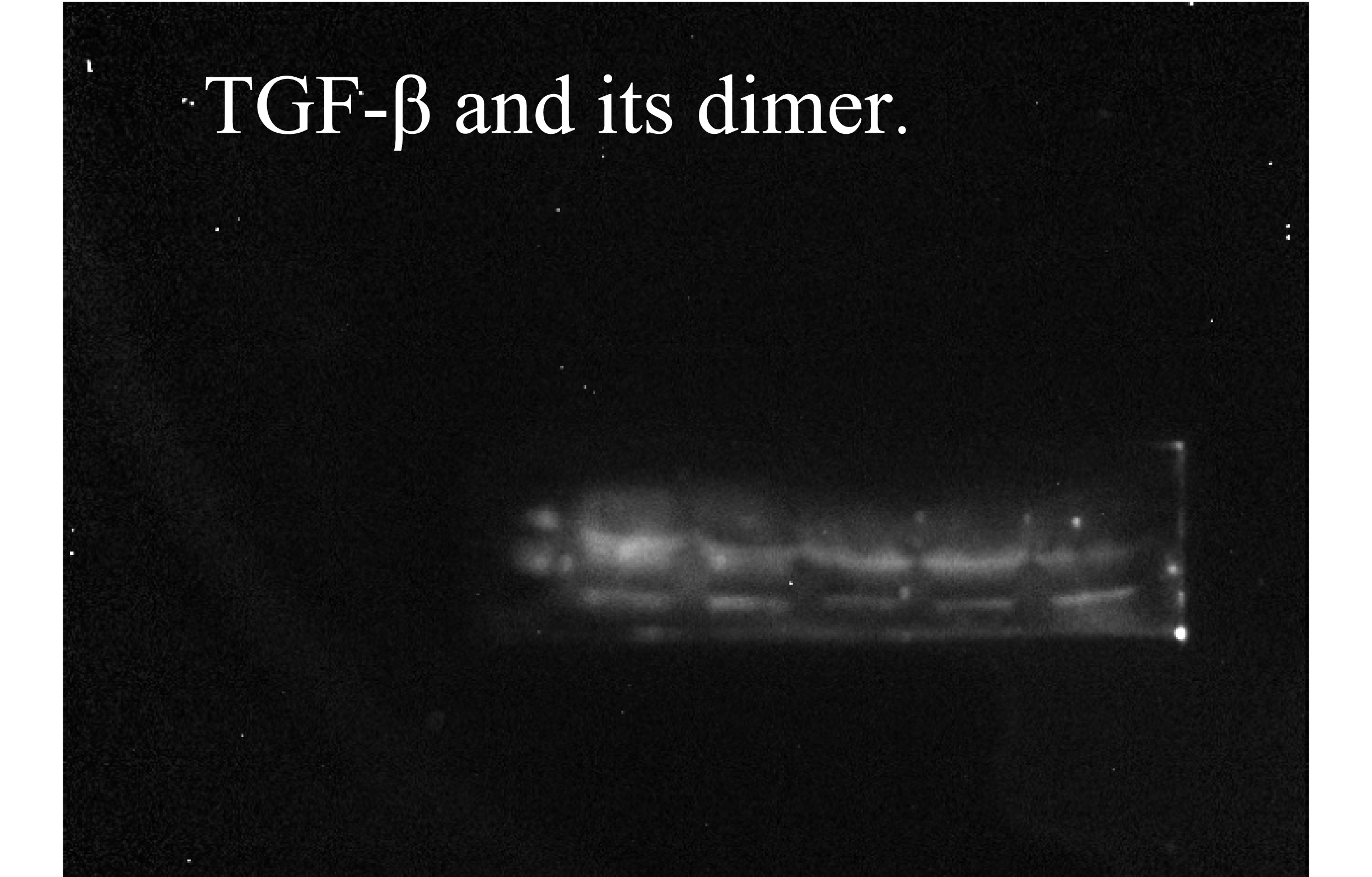

Supplement: Supplementary file 1 [file marinedrugs-21-00389-s001.zip › marinedrugs-2454142-supplementary/Original Images/TGF-a┬.tif]

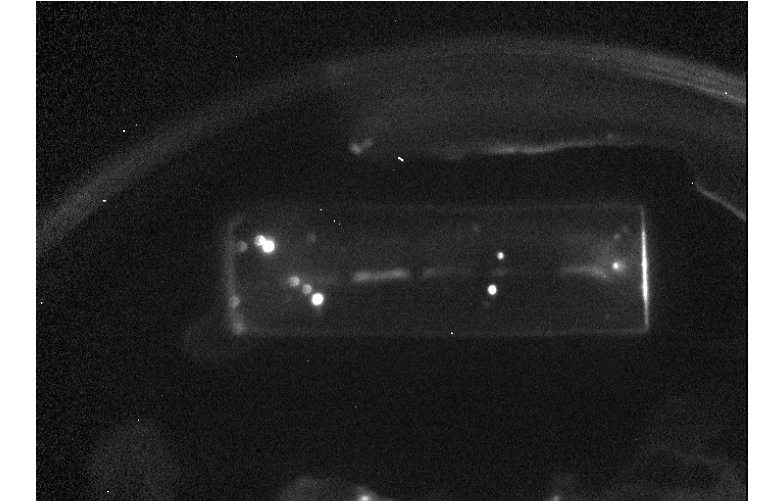

Supplement: Supplementary file 1 [file marinedrugs-21-00389-s001.zip › marinedrugs-2454142-supplementary/Original Images/TNF-a┴.tif]

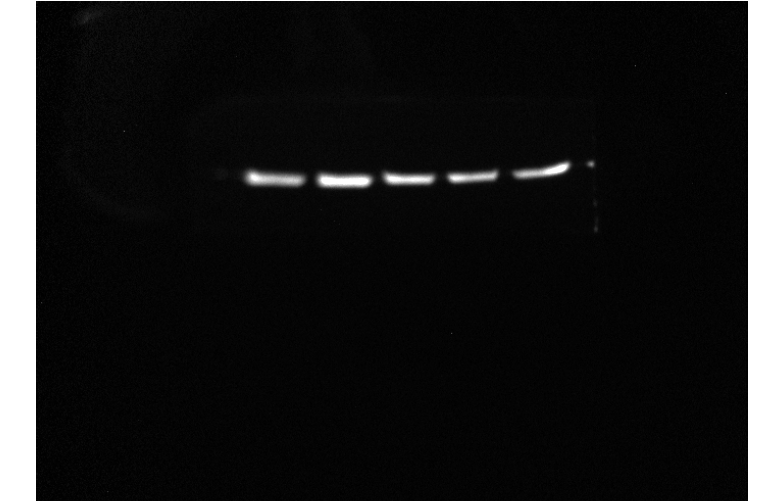

Supplement: Supplementary file 1 [file marinedrugs-21-00389-s001.zip › marinedrugs-2454142-supplementary/Original Images/a┬-actin.tif]
